# Systemic Bacterial Infection in a Captive Agouti (*Dasyprocta leporina* Linnaeus, 1758)

**DOI:** 10.1155/2022/8300247

**Published:** 2022-03-02

**Authors:** Kegan Romelle Jones, Kavita Ranjeeta Lall, Rod Suepaul, Gary Wayne Garcia

**Affiliations:** ^1^Department of Basic Veterinary Sciences (DBVS), School of Veterinary Medicine (SVM), Faculty of Medical Sciences (FMS), University of the West Indies, Mt. Hope Campus, Mt. Hope, Trinidad and Tobago; ^2^Department of Food Production (DFP), Faculty of Food and Agriculture (FFA), University of the West Indies, St. Augustine Campus, St. Augustine, Trinidad and Tobago

## Abstract

The agouti (*Dasyprocta leporina*) is a neotropical rodent which has the potential to be domesticated. As such, some research studies have been done on the biology of this animal. Recently, these animals are being kept in captivity as a source of animal protein. Animals which are kept in captivity may present diseases that would not have been reported in the wild due to lack of observation or the lack of occurrence. The aim of this short communication is to report a case of systemic bacterial infection that affected the lungs and liver of a captive agouti. Bacterial analysis revealed that the infection was caused by *Escherichia coli*. Bacterial infections have been reported in the mammary tissue as well as the skin of the agouti, but to the authors' knowledge, this is the first report of systemic infection in the agouti affecting several organs. This case was seen in a nine-month-old male agouti that was being housed at the University of the West Indies Field Station (UWI, UFS). The animal showed no apparent sign of disease except for lethargy and subsequently died before any treatment was administered. These findings showed that the agouti may have been under some stress (nutritional or environmental) which predisposed this animal to this infection. Future work has to address the nutritional requirements for the growing agouti as well as some treatment options for managements of similar cases in the future.

## 1. Introduction

The agouti (*Dasyprocta leporina*) is a neotropical rodent that has many functions in the wild and in captivity. In the wild, it provides seed dispersion for a variety of trees [1, 2], whilst in captivity, it can be used for educational and nutritional purposes. In many rural neotropical communities, these animals are a source of income and food with meat sharing practiced amongst some hunters [3, 4]. Recently, these animals are being held in captivity for farming purposes [5]. As such, more research is being conducted on the biology and the maintenance of these animals in captivity. The agouti is a medium-sized rodent (2-3 kg) that produces two offsprings per parturition [6] with a gestation period of 103 days [7]. These animals have an omnivorous diet [8–11] and are able to consume agricultural by-product [12]. Young animals can be weaned at one month and can reach market weight at nine months [13]. The ability to utilize local agricultural by-products make the agouti an excellent candidate for farming. The meat produced by the carcass is highly nutritiyous [14] with a high dressing percentage [15]. These animals also do not require an anthelmintic program once on a proper diet which reduces the cost of production [16].

However, problems can occur in rearing these animals in captivity that may not have occurred or be recorded in the wild. One such problem is disease. This area has recently been investigated with infectious and noninfectious diseases. Some authors have investigated infectious diseases such as parasites, bacteria, and viruses that may infect these animals. However, it was noted with many cases of infection with bacterial and parasitic organisms, the agouti was clinically healthy [17, 18]. There have also been bacteria found in the respiratory, gastrointestinal, and reproductive tracts of these animals that were part of their normal microflora [19, 20]. In contrast, Jones et al. [21] recorded mastitis in the agouti that was caused by *Staphylococcus aureus*, and Filgueira and others [22] reported a case of pododermatitis caused by *Corynebacterium pseudotuberculosis.* With limited published work on bacterial infections in the agouti, the aim of the current study was to present a case of bacterial infection in a captive population in Trinidad. This case was an animal which was raised in captivity at the University of the West Indies Field Station Farm located in Mt. Hope, Trinidad.

## 2. Case Report

### 2.1. History

A juvenile male albino agouti, part of a population held at University of the West Indies Field Station Farm, UWI, UFS, and weighing approximately 1 kg, was found to be lethargic. This animal was reared on concrete floor pens with ten other animals present in the enclosure. The diet of these animals consisted of locally available fruits and vegetables and supplemented with rabbit pellets (Mastermix ^®^). The enclosure of the animals was cleaned on a daily bases, and the animals were given water ad libitum. This animal was taken and placed into an individual cage for monitoring and possible testing and treatment. Within twelve hours of isolation, the animal died before testing or treatment was undertaken. Upon death, the animal's carcass was taken for postmortem examination, necropsy, histopathology, and bacteriological examination. All samples and analysis were done approximately four hours postmortem based on rigor mortis. The carcass when opened showed no signs of imbibition which can occur with necrosis as well as gut spillage. The gastrointestinal tract was intact when the necropsy was performed and samples taken.

### 2.2. Postmortem Examination

**Figure 1:**
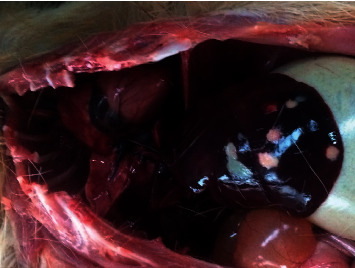
Liver of the agouti showing white and red circumscribed lesions.

**Figure 2:**
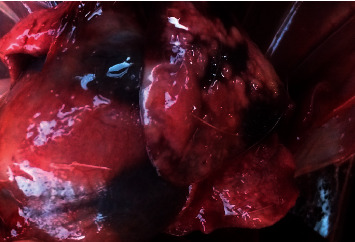
Lungs showing mottled appearance.

Grossly, the animal was in good body condition. Multiple smooth, friable, pale foci ranging from 10 to 20 mm in diameter occupied 5–10% of the liver (Figure 1). These lesions were distributed throughout the parenchyma of the liver. In the lungs, there were multiple irregular dark red areas of atelectasis occupying approximately 15–20% of the parenchyma (Figure 2).

### 2.3. Histopathological Examination

There were multiple areas of necrosis in the liver with large numbers of degenerate and intact neutrophils and macrophages at the edges and in surrounding tissues. Gram-negative bacteria were seen in the necrotic and inflamed tissue in the lung and the liver. Similar areas of necrosis and inflammation were scattered throughout the lungs. There was moderate to marked interstitial inflammation in the surrounding viable lung tissue.

### 2.4. Bacteriological Examination

Samples of the lesions were taken from the lungs and liver for bacteriological examination. Swabs taken from the lesions were inoculated onto Columbia agar supplemented with 5% sheep blood and MacConkey agar and incubated at 37°C for 18–24 hours in air. Biochemical tests as well as colony morphology were used in the identification of the bacteria. Some of the biochemical tests performed included citrate, coagulase, gas production from glucose, Gram stain, hydrogen sulphide production, hemolysis, indole, motility, methyl red, catalase, nitrate reduction, oxidase, triple sugar iron agar, urease, and sorbitol fermentation. Impression smears were also prepared from the swab with physiological saline on a glass slide. Following staining by Gram's technique, examination under light microscopy revealed individual colonies that were Gram negative. On the culture plates, pure cultures with light growth were obtained. The following results were obtained from the biochemical test performed on the pure culture: Gram stain negative, catalase positive, no growth on Simmons citrate, coagulase, oxidase and urease negative, gas produced from glucose, negative for hydrogen sulphide production, no hemolysis of sheep red blood cells, indole and methyl red positive, nitrate reduced, no pigment production on sheep blood agar, acid and gas produced in triple sugar iron agar, and acid produced from sorbitol. Based on these results, the isolate was identified as *E. coli*.

## 3. Discussion

The information obtained from diagnostic tests from the adult male albino revealed that the animal had a severe bacterial infection that affected the lungs and the liver. Nondomesticated animals tend to show little clinical signs to disease, which was the case with this agouti [23, 24]. There are few reported cases in the literature of infectious organisms affecting the agouti. In most cases, the agouti serves as a reservoir host for a variety of diseases without showing any clinical signs. Some of the associated organisms are *Toxoplasma gondii* [25–27], *Leishmania* spp. [28, 29], *Trypanosoma cruzi* [30, 31], and *Echinococcus oligarthrus* [32]. However, clinical cases of bacterial diseases have been rarely reported.

Bacterial microflora of the eye, respiratory tract, gastrointestinal tract, and reproductive tract of the agouti have been described [19, 20, 33, 34]. Some of these bacterial species included *E. coli*, *Streptococcus* spp., *Hafnia alvei*, *Bacillus* spp., *Staphylococcus epidermidis*, *S. intermedius*, *Salmonella* spp., *Campylobacter* spp., *Klebsiella pneumoniae*, *Pseudomonas aeruginosa*, *Citrobacter diversus*, and *Acinetobacter calcoaceticus* [19, 20, 33, 34]. It should also be noted that antibody titres for *Leptospira* and *Brucella* species have been found in wild caught agouti [26, 35]. This shows that the agouti can serve as a reservoir host for the spread of leptospirosis and brucellosis.

In the current case, the infectious organism was identified as *E. coli*, a common commensal of the gastrointestinal tract. This organism was found in the lungs and liver, which would suggest a systemic infection, and the animal may have been in a state of sepsis. There are a few cases of bacterial diseases affecting the skin and mammary tissues of agouti [21, 24]. The reported skin disease may have been through an initial traumatic event and subsequent seeding of *C. pseudotuberculosis*. The only other reported case of bacterial infection in the agouti was in the mammary system which was invaded by *S. aureus* [21].

The systemic invasion of *E. coli* in this case may be due to some stressors (environmental or nutritional) which decreased the animal's immune response. In the management of the agouti, the specific nutritional requirements for the agouti are unknown. John and Jones [11] showed that agouti can maintain their adult bodyweight on a 17% crude protein diet. However, the protein requirement for growing (juvenile) animals is currently unknown, and the locally available fruits and rabbit ration supplementation may not have adequate nutrients for this physiological state. Environmental stressor that affects the agouti in the wild mostly will occur from predators [36]. However, in captivity, this type of stressor will not be experienced. However, stressors may occur from pen mates as this group had juveniles, and there were both males and females in the enclosure. The order of dominance in the pen may have caused stress in docile individuals leading to an immunocompromised state.

Systemic bacterial infections have been reported in many domesticated rodents where there is knowledge of appropriate diagnostic tests and treatment that can be performed [37]. The causative agent can be identified through a biopsy of the infected tissue (in this case the liver) and standard bacteriological testing done [37]. The treatment of this infection would have been through the use of an appropriate antibiotic, having consideration to avoid dysbiosis of the normal flora of the gastrointestinal tract of rodents which are hindgut fermenters [37].

## 4. Conclusion

The information presented above showed pneumonia and hepatitis caused by *E. coli*, a part of the normal gut flora. However, in animals with an immunocompromised state, this organism can cause systemic infection. The animal died suddenly in this case, but in future cases, diagnosis can be confirmed via biopsy of the affected organ with bacteriological testing. Treatment options would include appropriate antibiotic therapy with supportive treatment. It should be noted that this case may be related to high levels of stress (nutritional or environmental). Therefore, captive nondomesticated animals such as the agouti should have decreased stressed levels with adequate nutrition and housing.

## Data Availability

The data used to support the findings of this study are included within the article.
